# Platelet Subtypes in Inflammatory Settings

**DOI:** 10.3389/fcvm.2022.823549

**Published:** 2022-04-07

**Authors:** Muataz Ali Hamad, Krystin Krauel, Nancy Schanze, Nadine Gauchel, Peter Stachon, Thomas Nuehrenberg, Mark Zurek, Daniel Duerschmied

**Affiliations:** ^1^Department of Cardiology and Angiology I, Heart Center, Faculty of Medicine, University of Freiburg, Freiburg im Breisgau, Germany; ^2^Spemann Graduate School of Biology and Medicine (SGBM), University of Freiburg, Freiburg im Breisgau, Germany; ^3^Faculty of Biology, University of Freiburg, Freiburg im Breisgau, Germany; ^4^Department of Cardiology, Angiology, Haemostaseology, and Medical Intensive Care, University Medical Centre Mannheim, Medical Faculty Mannheim, Heidelberg University, Mannheim, Germany; ^5^Department of Cardiology and Angiology II, Heart Center, Faculty of Medicine, University of Freiburg, Bad Krozingen, Germany; ^6^European Center for AngioScience (ECAS) and German Center for Cardiovascular Research (DZHK) Partner Site Heidelberg/Mannheim, Mannheim, Germany

**Keywords:** platelets, reticulated platelets, procoagulant platelets, vascular, immunology, inflammation

## Abstract

In addition to their essential role in hemostasis and thrombosis, platelets also modulate inflammatory reactions and immune responses. This is achieved by specialized surface receptors as well as secretory products including inflammatory mediators and cytokines. Platelets can support and facilitate the recruitment of leukocytes into inflamed tissue. The various properties of platelet function make it less surprising that circulating platelets are different within one individual. Platelets have different physical properties leading to distinct subtypes of platelets based either on their function (procoagulant, aggregatory, secretory) or their age (reticulated/immature, non-reticulated/mature). To understand the significance of platelet phenotypic variation, qualitatively distinguishable platelet phenotypes should be studied in a variety of physiological and pathological circumstances. The advancement in proteomics instrumentation and tools (such as mass spectrometry-driven approaches) improved the ability to perform studies beyond that of foundational work. Despite the wealth of knowledge around molecular processes in platelets, knowledge gaps in understanding platelet phenotypes in health and disease exist. In this review, we report an overview of the role of platelet subpopulations in inflammation and a selection of tools for investigating the role of platelet subpopulations in inflammation.

## Introduction

Platelets promptly initiate a set of responses at the endothelium upon encountering molecular or biophysical cues of aberrations in vascular flow, form, or function. Such responses include platelet adhesion to endothelium, shape change, secretion, and aggregation which is physiologically critical to limit vessel leakage and prevent bleeding (1, 2). There are roughly 300,000 platelets per μl of blood, with a cell volume of 7 fl and a mean surface area of 8 μm^2^, which makes them display a larger total volume and surface area compared to all other leukocyte subtypes. Platelet involvement in inflammatory or immune processes *via* their proinflammatory mediators as well as surface receptors clearly shows that they have a role that exceeds being mere players in hemostasis and thrombosis. Thrombus formation can be divided into 3 distinct phases: adhesion, activation, and aggregation of platelets (3). Upon activation, platelets release considerable quantities of secretory products and express a multitude of immune receptors on their membrane giving them the ability to support the recruitment of leukocytes into inflamed tissue and regulate their function. Platelets are able to form aggregates with neutrophils (Platelet-Neutrophil Complexes, PNCs), which leads to mutual activation of both cells resulting in cytokine release, exposition of certain adhesion molecules, and receptors on the cell surface which in turn facilitates extravasation of these cells into inflamed tissue (4). The PNC formation is mainly mediated by the platelet’s P-selectin (CD62P) and its ligand P-selectin glycoprotein ligand-1 (PSGL-1) on neutrophils (4, 5). The importance of the P-selectin/PSGL-1 axis has been shown, as blocking platelet CD62P could abolish PNC formation in murine and human whole blood samples (6). Hence, platelets provide an ideal and crucial link to explain the inseparability of thrombotic and inflammatory events such as atherosclerosis or atherothrombosis. Circulating platelets differ one from another with respect to their (a) size (7, 8), (b) surface receptor expression (9–11), (c) glycosylation (12), (d) granule content (13, 14), (e) response to agonist stimulation (15–17), and (f) participation in thrombus formation (18), meaning that within the normal platelet pool there are some distinct subpopulations each performing a certain role in different settings. Indeed, in contrast to rapid shape change and other responses platelets can also undergo more extended transitions in phenotype that are increasingly associated with chronic disease (1, 19, 20). The phenotype in its generalized concept refers to the observable, distinguishable, or measurable type of phenomenon exhibited by a biological entity resulting from the interaction of its genotype and environment (21, 22). The notion to describe single-cell properties of platelets or platelet subpopulations that deviate from normal is gaining more attention to evaluate whether these phenotypes are indicative or causative agents of disease. Both *in vitro* as well as *in vivo* studies have begun to catalog heterogeneous subpopulations of platelets described as procoagulant, “angry,” coated, secretory, exhausted, or sticky – in different pathological settings. Despite the wealth of knowledge around molecular processes in platelets, knowledge gaps in understanding platelet phenotypes in health and disease exist. Here, we present an overview of different platelet phenotypes and their behavior during an inflammatory response.

## Platelet Subtypes

**FIGURE 1:**
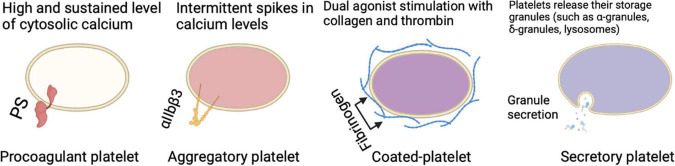
Different circulating platelet subtypes each perform a certain role in different settings and participation in thrombus formation.

Platelets have different physical properties leading to distinct subtypes of platelets based either on their function (procoagulant, aggregatory, secretory) (Figure 1) or their age (reticulated/immature, non-reticulated/mature). Indeed, it has been described that at wound sites there is a subpopulation that is the first to adhere to collagen and spread to form a monolayer known as “vanguard platelets” and a second population that adheres to and spread onto nearby collagen or over the vanguard platelets described as “follower platelets” (23). When “vanguard platelets” adhere to collagen, they rapidly begin to spread and lose the distinctive mound-shaped structure. Then, this process is usually followed by additional adhesion of vanguard platelets as well as other platelets (follower platelets) (23). The platelet-platelet interactions are crucial for follower platelets deposition thus, functional GPIIb/IIIa receptors are indispensable. From another perspective, another platelet subpopulation lacks endothelial nitric oxide synthase (eNOS), fails to produce nitric oxide, and has a down-regulated soluble guanylate cyclase signaling pathway. In turn, this subpopulation of platelets shows greater activation of αIIbβ3 and adhesion to collagen, resulting in larger aggregates than eNOS-positive platelets (24). In the concept of heterocellular control of coagulation, platelets can be distinguished in different tasks such as control of thrombin generation, support of fibrin formation, and regulation of fibrin clot retraction (25). Within the functional scope, there are two distinct phenotypes of platelets with distinct surface properties facilitating these coagulant functions. One is a phenotype that externalizes phosphatidylserine (PS) and binds tenase and prothrombinase complexes, leading to accelerated coagulation at the wound site and controlling thrombin and fibrin generation (25). A second phenotype is characterized by active integrin αIIbβ3, which tightens the clot into an impermeable cell mass by pulling fibrin over the platelet plug (25). The youngest platelet subtype released into the circulation appears to be more reactive and shows an increased tendency to recruit other platelets and immune cells to the site of injury. The newly formed platelets contain a residual amount of the megakaryocytic messenger RNA (mRNA) that gives them a greater array of functional pathways (26). As platelets age, the total protein content is degraded or lost without the possibility for replacement leaving old platelets with several biological alterations in function (26). Differences related to platelet age propose a young platelet subpopulation that are rapid hemostatic responders and an old platelet population with higher apoptosis and senescence. Some data also showed that lung megakaryocytes (Mks) have immune cell characteristics that differ from bone marrow (BM) Mks characterized by antigen-presenting-cell-like cell markers and functions (27). These site-specific cell characteristics may in part be driven by the tissue environment as lungs and BM are very different tissue environments. In the BM, Mks face a few pathogen challenges and the environment is relatively hypoxic, while in the lung there’s high oxygen (O_2_) as well as a microbiome. Such immune regulatory functions of Mks described here are likely to be forwarded to the platelet progeny. It is clear that there are intrinsic platelet factors (such as platelet size and structure, protein composition, genetic factors, and platelet age), and environmental factors (such as the local rheology, exposure to agonists, surrounding cells, and plasma) that account for the response heterogeneity. The evidence that supports the concept of functionally different subpopulations of platelets is well-reported and targeting platelet subpopulations might be an encouraging antithrombotic approach.

### Procoagulant and Aggregatory Platelets

There are major differences between aggregatory and procoagulant platelets which leads to the question of how a platelet becomes procoagulant while another does not. For a platelet to become procoagulant, it is required to have a high and sustained calcium rise leading to PS externalization, coagulation factor binding, and calpain-mediated inactivation of α_IIb_β_3_ integrin (23, 28–30). The fundamental calcium rise for the procoagulant response is led by calcium mobilization from intracellular stores, which is associated with the activation of calcium (Ca^2+^) activated chloride channels, resulting in an initial salt entry, which is then followed by the influx of water (23, 25, 31, 32). The electrochemical drive for Ca^2+^ entry is enhanced as well as membrane hyperpolarization as a result of the chloride ion entry, and that’s achieved through both store-operated and store-independent pathways (23, 29, 31–33). Jointly these responses guarantee a high and sustained level of cytosolic calcium required to drive the procoagulant response. It is important to emphasize that the irreversible membrane swelling or ballooning that results from the physical disruption of the membrane-cytoskeleton interaction and an increase in internal hydrostatic pressure provided by a coordinated fluid entry system is a key event during procoagulant platelet formation (23). All these changes lead to a distinct population of highly activated platelets characterized by surface-exposed PS, prolonged cytosolic Ca^2+^ rises, a rounded structure, and the ability to bind coagulation factors such as factor V (FV) and factor X (FX) (25). Meanwhile, a different pattern of calcium signaling is found in aggregate-forming platelets, which is rather characterized by intermittent spikes in calcium levels or oscillatory calcium responses (18, 23, 29, 34). Aggregatory platelets have active α_IIb_β_3_ integrins on their surface which is a major difference to PS-exposing platelets enabling them to consolidate the plug by clot retraction (25). This might be seen as a mechanism for narrowing the gaps between platelets to allow contact-dependent signaling (35). Upon dual agonist stimulation of platelets with collagen and thrombin, a subpopulation of cells is observed known as coated-platelets (formerly known as COAT-platelets), which retains high levels of several procoagulant proteins on its surface resulting in an unparalleled ability to promote thrombin generation (36).

### Secretory Platelets

Besides the procoagulant and aggregatory roles that platelets play, upon activation, platelets act as secretory cells. Platelets contain multiple storage granules (such as α-granules, δ-granules, and lysosomes) that release their content when activated by fusing the intracellular granules with the plasma membrane. Besides the intracellular vesicles, platelets are able to produce extracellular vesicles, these secretions in turn can influence many physiological and pathophysiological processes. The importance of platelet secretion granules and their content (such as growth factors, chemokines, cytokines, and microbicidal proteins) can be further elucidated by looking at platelets lacking α-granules (such as in gray platelet syndrome), δ-granules (such as in Hermansky-Pudlak syndrome), or both can result in bleeding, reduced inflammation, and impaired vascular remodeling and wound healing (37). The extracellular vesicles in turn which can be further classified to exosomes and microvesicles also seem to play a role in blood-related processes (38). In the context of inflammation, platelet-derived extracellular vesicles interact with leukocytes and their inflammatory role can be observed in rheumatoid arthritis stimulating cytokine production from synovial fibroblasts (37). Not only they are able to secrete multiple products, but platelets are also able to take up plasma-derived or cell-derived components such as RNA species from tumor cells (39). Taken together, all of these multiple mechanisms indicate that there is bidirectional communication of platelets and platelet-derived mediators with components of the inflammatory pathways, in a manner that platelets influence their environment, and their environment in return has an influence on them. This concept can be further supported by the observation of platelets interaction with leukocytes and the coagulation system during thromboinflammation. The so-called “exhausted platelets” which is a phenotype seen in patients with solid tumors, sepsis, or stroke, characterized by low platelet activation responses *in vitro*, is also another example of how the environment affects platelets (40).

### Young and Senescent Platelets

**FIGURE 2:**
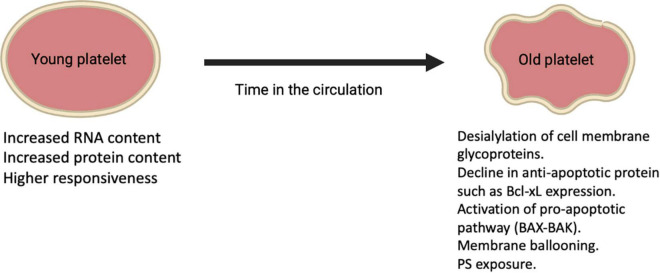
Alterations on platelets during their lifetime in the circulation.

Platelets are anucleate cells that circulate for approximately 7–10 days during which their protein composition change as they age leading to alterations in structure and function. Reticulated platelets (RP) (also known as immature platelets) represent the youngest platelets released into the circulation from Mks and are referred to as “reticulated,” analogous to reticulocytes in erythropoiesis (41). These young platelets appear to have increased RNA content compared to mature platelets as well as more dense granules and higher levels of surface activation markers upon stimulation (Figure 2) (37). Hence, this platelet fraction is considered to show increased reactivity and is associated with impaired response to antiplatelet therapy (42–44). RP is about 2–3 times higher in the BM compared to peripheral blood where they are present for ≤24 h in humans and count for around 12% of the total platelet population in a steady-state (45–47). Platelet aging is linked to a decrease in cytoskeletal protein, lower mitochondria number, as well as lower calcium dynamics and granule secretion (26). A recent study showed that the total protein content was almost 50% lower in old platelets compared to young platelets (26). Besides, during conditions with increased platelet turnover, RP appear to be larger than mature platelets, for instance, in humans after chemotherapy (48). On the other hand, platelet size may not correlate with platelet age under steady-state platelet production and clearance further confirmed using the Abbott Sapphire analyzer showing a negative association between RP and mean platelet volume (MPV) (49). Despite the fact that platelets are anucleate, they still share some similarities in mechanisms that are used by nucleated cells for programmed cell death resulting in a steady state of platelet production and clearance in health. With aging in the circulation, platelets appear to show a gradual decline in Bcl-xL expression, which is an anti-apoptotic protein that in turn liberates the proapoptotic Bak/Bax proteins leading to Bak/Bax pathway activation and starting mitochondrial-dependent apoptosis and subsequent PS exposure (25). After PS exposure on their outer membrane surface, platelets are cleared *via* scavenging receptors on phagocytic cells in the liver and other organs. The apoptotic PS exposure differs mechanically from that of agonist-induced, as apoptotic PS exposure appears to rely on caspase activation (25). These clear distinctions between apoptotic and agonist-stimulated PS-exposing platelets have led to the suggestion that the latter are activated by a necrotic cell death pathway. The loss of the negatively charged sugar moiety sialic acid from the surface of senescent platelets is another way by which platelets are cleared from the circulation by the hepatic asialoglycoprotein receptor 1 (76).

## Platelet Subpopulations in Cardiovascular Diseases

In the scope of cardiovascular diseases (CVD), different platelet subpopulations have different roles in the prognoses of the disease, and some of them are linked to a higher risk of major adverse cardiovascular events and death (48, 50). While the procoagulant activity of platelets is vital for hemostasis after vessel injury, it has been linked to stroke and coronary artery disease (51–53). Indeed, stable coronary artery disease (CAD) has been associated with a heightened procoagulant platelet response when compared to healthy controls, and this response is not even inhibited by aspirin alone (54). High levels of coated platelets were also associated with an increased risk for recurrent infarction in non-lacunar stroke (55). RP might have a significant role in myocardial ischemia/reperfusion (I/R) injury, caused by the interventional reopening of an occluded coronary vessel in the context of myocardial infarction (MI) especially as RP seem to exhibit resistance to common antiplatelet therapies at least to some extent (56). Beyond providing therapeutic targets, measuring these heterogeneous subpopulations of platelets with specific molecular properties may offer the means to define, predict and diagnose platelet-associated conditions – especially vasculopathy that is progressed by inflammatory, procoagulant, and other platelet responses.

## Platelet Subpopulations in Infectious Diseases

In infections, the formation of an intravascular thrombus might be part of the process of pathogen containment which is also known as “immunothrombosis,” and platelets are key players in promoting this process. Although platelets and their products suppress infection, during an infection platelet consumption and removal are increased often leading to thrombocytopenia. Platelets can be immunomodulatory cells during an infection regulating and/or participating in the inflammatory response with certain dysregulation in platelets subpopulation such as higher levels of young/reticulated platelets which can be reported as high immature platelet fraction (IPF) levels during infection. For instance, during dengue infection thrombocytopenia is a common complication and IPF can be used as an indicator to predict platelet recovery 24–48 h earlier (57). Another example is the significant correlation between higher IPF and the diagnosis of sepsis as well as a predictor of severe thrombocytopenia and mortality (43). One more example is the COVID-19 caused by the severe acute respiratory syndrome corona virus-2 (SARS-CoV-2), which is associated with a high incidence of venous and arterial thromboembolic events and the pathophysiology seems to be multifactorial. During COVID-19 infection platelets express procoagulant phenotype, which shows enhanced PS externalization and increased apoptotic markers (58, 59). There’s a strong need for markers to guide antithrombotic therapy in COVID-19 patients and to somehow monitor the platelets’ dynamics. IPF provides indices of platelet turnover and reactivity in patients with COVID-19 respiratory disease which might serve as a prognostic marker for disease severity. Several studies have shown that patients with COVID-19 had higher levels of IPF and immature platelet count (IPC) than healthy controls and patients with cardiovascular risk factors (60–64). IPF has been a useful tool not only in detecting an infectious state, but also in differentiating a serious state from a minor one (65). Such findings suggest that platelets are refractory to the inflammatory process that is happening which makes them (platelet population as whole or subpopulation markers) a very good candidate to be used as a diagnostic/prognostic marker in certain conditions such as vascular diseases, cancer, infectious diseases (65), pregnancy complications, liver diseases (66–68). More recent findings on the immunomodulatory role of platelets during an infectious status are the platelet’s role during the hand, foot, and mouth disease (HFMD) caused by enterovirus 71 (EV71). It has been shown that platelets have distinct roles in the pathogenesis of HFMD by regulating the pathogenic CD4 + T cell differentiation and function (69). When exploring the mechanism by which platelets regulate CD4 + T cell differentiation, gene expression of the T cell surface molecule CD40 was found to be decreased in the mild group of patients while it increases gradually in the severe group. PSGL-1 gene expression on the other hand, which binds to the platelet’s P-selectin was also found to increase significantly in the severe group. Such findings suggest that platelets in severe patients with HFMD mainly regulate T cells through CD40L, GPIbα, and CD62P. Not only that, but plateletcrit and platelet count levels both were positively associated with the severity of HFMD (69). Alongside the change in the total platelet count, platelet subpopulations would also show certain trends of dysregulation during different disease states.

## Tools to Investigate Platelet Subsets

Advances in research methodology and technology such as the application of flow cytometry to platelet studies have enhanced our ability to study platelet subpopulations. The platelets are stained with receptor-specific monoclonal antibodies conjugated to fluorescent probes and evaluated with fluorescence flow cytometry (FFC). FFC offers the possibility to evaluate platelets and their function in small blood volumes and very rapidly (∼10,000 platelets/min), and hence, FFC has been traditionally used in clinical and research settings (70, 71). In terms of platelet subpopulations, certain markers could be applied to distinguish them and evaluate their dynamics. For instance, young/reticulated can be evaluated and differentiated from the older platelets in the circulation depending on their RNA content using nucleic acid dyes. Thiazole orange or SYTO13 both have been reported as indicators of reticulated platelets (72, 73). Another method to evaluate reticulated platelets is using automated hematology analyzers such as the Sysmex analyzers reported as IPF% or #IPC (74). There are several pre- and post-analytical considerations when evaluating platelets using these techniques and we have reviewed all these considerations in greater detail in a previous review which readers might refer to (41). Annexin V could be used in FC to report the levels of procoagulant or apoptotic platelets due to its ability to bind to PS (75). Aggregatory platelets could also be evaluated using antibodies against the active αIIbβ3 integrins. While desialylated platelets can be detected by conjugating the ricinus communis agglutinin I (RCA-1) lectins with a fluorochrom, which specifically target exposed galactose residues following GP desialylation (76). FFC comes with a major limitation which is the limited number of parameters that can be simultaneously analyzed due to emission spectra overlap increasing the complexity of the compensation required for accurate analysis (77). The way to solve the inherited limitation of FFC is to overcome the spectral overlap and have the possibility to evaluate different markers simultaneously on individual platelets. One way to achieve that is by applying mass cytometry (MC), which is a next-generation flow cytometry platform and using probes that are conjugated to heavy metal isotopes instead of fluorescent dyes and time-of-flight as a detection technique (78, 79). Using MC there will be no need for compensation as this detection technique has minimal spectral or channel overlap resulting in an increase in the number of cellular parameters that can be analyzed simultaneously on individual cells. MC enables simultaneous phenotypic and functional analysis of multiple parameters applying panels of up to 45 different cellular parameters (80), and in theory up to 100 different parameters (81, 82). The possibility to evaluate multiple markers can be a great way to evaluate platelet heterogeneity within the platelet pool of healthy donors and patients. Platelets could be evaluated in whole blood or in the form of platelet-rich plasma (PRP), which allows the evaluation of high numbers of events enabling the investigation of previously unappreciated small platelet subgroups. One of the limitations with platelet subsets studies, in general, is the lack of standardized protocols that are easily reproducible. Recently, a structured method to stain and evaluate platelets from PRP using CyTOF was published which allows the acquisition of 300,000 to 500,000 events and recording the expression of up to 40 markers at once (78). MC data can be analyzed using Visual stochastic neighbor embedding (viSNE) to visualize high-dimensional single-cell data, for platelet-specific analysis some groups have developed freely available analysis pipelines such as CYANUS (83). As expected, such a detailed evaluation of the platelet pool revealed some differences between baseline and stimulated samples in healthy donors. For instance, studies have shown that the expression of CD42a and CD42b receptors goes down after TRAP stimulation (84). MC analysis of platelets from Glanzmann Thrombasthenia (GT) patients show a significant reduction in CD41, CD61, and activated integrin αIIbβ3 surface expression (84).

## Therapeutic Targeting of Platelet Subpopulations

Dual antiplatelet therapy is recommended for secondary prevention of coronary artery disease, including a cyclo-oxygenase-1 inhibitor, and a platelet adenosine diphosphate (P2Y_12_) receptor inhibitor (85). Aspirin is a dose-dependent cyclooxygenase (COX) inhibitor that inhibits COX-1, and at higher concentrations, it inhibits COX-2, through irreversible acetylation of a serine residue in the catalytic channel (86, 87). This inhibition will translate into permanent suppression of thromboxane A2 (TXA2) generation in platelets. On the other hand, inhibiting the P2Y_12_ receptor on platelets prevents platelet activation by ADP. There are several P2Y_12_ inhibitors such as clopidogrel which has an antiaggregatory effect and is beneficial in the treatment of MI. Subpopulations of platelets exist as a result of variability in surface molecules expression which might be attributed to differences related to platelet age or differences in exposure to local *in vivo* activating conditions. The activated procoagulant platelets come with unique challenges for drug therapy such as aspirin and P2Y_12_ blockers that usually target the inhibition of platelet secretion, which in turn demonstrates a need for alternative targets. Now, with the advancement in mass spectrometry instrumentation, it is possible to perform quantitative studies beyond that of earlier work allowing the discoveries of the importance of every receptor or platelet state and possibly targeting them more beneficially. The existing literature suggests a need for a clinically effective antiplatelet-antiprocoagulant regimen to limit the procoagulant response of platelets. For instance, it has been observed that cyclic-adenosine-monophosphate (cAMP) elevation can sufficiently inhibit the initiation of COVID-19 antibody-mediated procoagulant platelet generation thus reducing subsequent thrombus formation (88). Indeed, inducing increased intracellular cAMP levels in platelets using clinically approved therapeutic agents such as iloprost was shown to prevent COVID-19 antibody-mediated coagulopathy. A different potential agent might be acetazolamide, which is a mild diuretic that is already in clinical use and has been shown as a potent antithrombotic (89). Acetazolamide is a carbonic anhydrase inhibitor that suppresses platelet procoagulant responses and thrombus formation by distinct mechanisms and is also capable of blocking water entry *via* the water channel aquaporin 1 (AQP1) (89, 90). Another attractive target for the development of new antithrombotic drugs would be the PAR1 system, which mediates human platelet activation at low thrombin concentration, unlike PAR4 which requires a higher concentration of thrombin for platelet activation and thus preserve a protective mechanism in situations such as trauma (91). Indeed, preclinical and early clinical work on PAR1 inhibition was promising in terms of safety profile and did not affect primary hemostasis. Vorapaxar (SCH530348), developed by Schering Plough is one of the anti-PAR1 molecules used in clinical trials (92). Another molecule is atopaxar (E5555), developed by Eisai pharmaceuticals. It is a small organic molecule, orally active, an inhibitor that binds at the tethered ligand binding site of PAR1 (93). An increase of bleeding events in the study group seems to be reported when compared to the placebo group. On the other hand, adding a third antiplatelet drug to the standard dual antiplatelet therapy is a higher risk of bleeding thus, these agents should be considered differently in future trials not only as an “add-on” therapy. Targeting primary platelet activation pathways is also one of the recent efforts to develop new classes of antiplatelet drugs. Targeting the immunoreceptor tyrosine-based activation motif (ITAM)-containing collagen receptor GPVI/FcRγ-chain complex would provide platelet inhibition due to the role of these receptors in the downregulation of platelet ITAM-receptor signaling (94). The results from targeting GPVI are encouraging with reduced aggregation and smaller arterial thrombi, with no major bleeding complications. It has been suggested in the literature that both PECAM-1 (which inhibit signaling downstream of the collagen receptor GPVI and other platelet activation pathways, such as those mediated by ADP and thrombin), and G6b-B (which inhibits platelet activation by the ITAM-bearing receptors GPVI and CLEC-2) are worthy of consideration as targets for new antiplatelet therapy. For greater details on targeting PECAM-1 and G6b-B as antithrombotic targets, readers may refer to (94).

## Conclusion

The responsive transitions in form and function platelets undergo are essential to repair vascular endothelium and mediate hemostasis. Platelets are central players in immunosurveillance and vascular inflammation as they facilitate the recruitment of leukocytes into the inflamed tissue as well as enhancing leukocytes’ contact with endothelium, which is achieved by the different adhesion molecules and soluble immune mediators. In response to a variety of physiological and pathological circumstances, qualitatively distinguishable platelet phenotypes are increasingly reported in the circulation with conceptually vague origins and significance. It is of great importance to have a meaningful and practical manner where platelets themselves can serve as important puzzle components and also provide physiologically relevant examples on cellular function and vascular wellbeing.

## Author Contributions

MAH and DD: conceptualization. MAH: methodology. All authors: writing—original draft preparation and writing—review and editing. KK and DD: supervision. All authors have read and agreed to the published version of the manuscript.

## Conflict of Interest

The authors declare that the research was conducted in the absence of any commercial or financial relationships that could be construed as a potential conflict of interest.

## Publisher’s Note

All claims expressed in this article are solely those of the authors and do not necessarily represent those of their affiliated organizations, or those of the publisher, the editors and the reviewers. Any product that may be evaluated in this article, or claim that may be made by its manufacturer, is not guaranteed or endorsed by the publisher.
